# Photoacoustic imaging for surgical guidance: Principles, applications, and outlook

**DOI:** 10.1063/5.0018190

**Published:** 2020-08-13

**Authors:** Muyinatu A. Lediju Bell

**Affiliations:** Department of Electrical and Computer Engineering, Johns Hopkins University, Baltimore, Maryland 21218, USA

## Abstract

Minimally invasive surgeries often require complicated maneuvers and delicate hand–eye coordination and ideally would incorporate “x-ray vision” to see beyond tool tips and underneath tissues prior to making incisions. Photoacoustic imaging has the potential to offer this feature but not with ionizing x-rays. Instead, optical fibers and acoustic receivers enable photoacoustic sensing of major structures—such as blood vessels and nerves—that are otherwise hidden from view. This imaging process is initiated by transmitting laser pulses that illuminate regions of interest, causing thermal expansion and the generation of sound waves that are detectable with conventional ultrasound transducers. The recorded signals are then converted to images through the beamforming process. Photoacoustic imaging may be implemented to both target and avoid blood-rich surgical contents (and in some cases simultaneously or independently visualize optical fiber tips or metallic surgical tool tips) in order to prevent accidental injury and assist device operators during minimally invasive surgeries and interventional procedures. Novel light delivery systems, counterintuitive findings, and robotic integration methods introduced by the Photoacoustic & Ultrasonic Systems Engineering Lab are summarized in this invited Perspective, setting the foundation and rationale for the subsequent discussion of the author’s views on possible future directions for this exciting frontier known as photoacoustic-guided surgery.

## I. INTRODUCTION

The physical principles that govern photoacoustic imaging technology are summarized in several review articles.^1–3^ The basic principle relies on the illumination of a region of interest with pulsed light that is then absorbed by photoabsorbers within the tissue (such as hemoglobin), resulting in a small mK rise in temperature due to vibrational and collisional relaxation. The temperature rise results in thermal expansion generating an acoustic pressure wave. This pressure wave can be received by an ultrasound transducer and reconstructed into an image.^2,3^ Contrast in photoacoustic images is primarily derived from differences in the optical absorption spectrum of different tissues.^3,4^ Because the light is incident on a specific region of tissue, the temperature rise and, therefore, the resulting pressure wave both depend on the optical absorption properties within the illuminated region of tissue. Specific targets for photoacoustic imaging include blood, the fat within the myelin sheath of nerves, and metallic tool tips.

Photoacoustic imaging system components generally include a light source (typically a nanosecond pulsed laser) and an ultrasound system capable of clinical grade images. Ultrasound images may also be used to provide anatomical structural context for the optical absorption maps provided by photoacoustic images. Because the same ultrasound sensor is used, ultrasound and photoacoustic images can be inherently co-registered to each other.

**FIG. 1.**
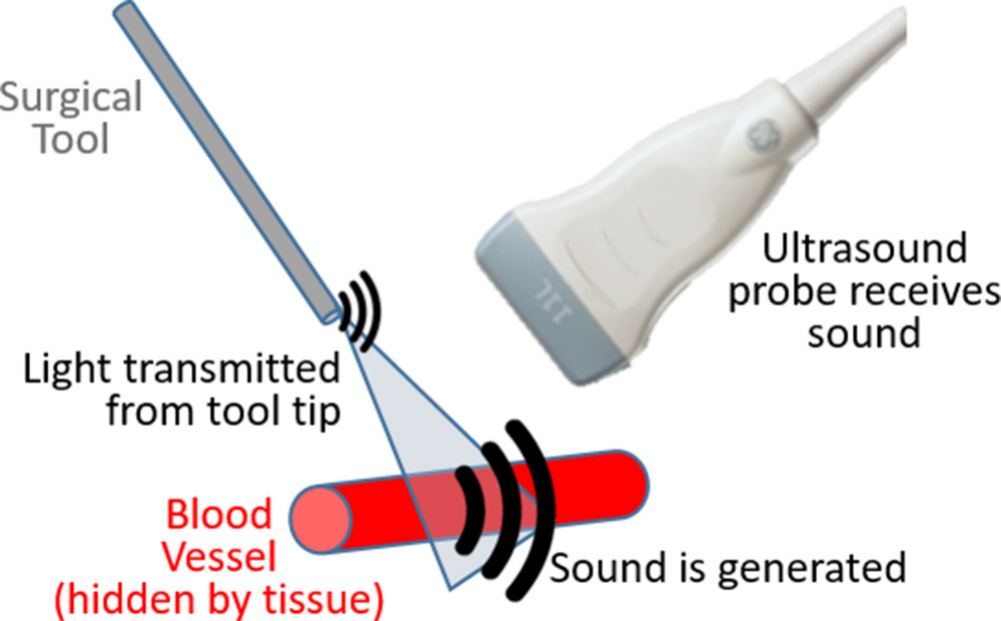
Concept of photoacoustic-guided surgery with intentional flexibility between light transmission and external sound reception.

**FIG. 2.**
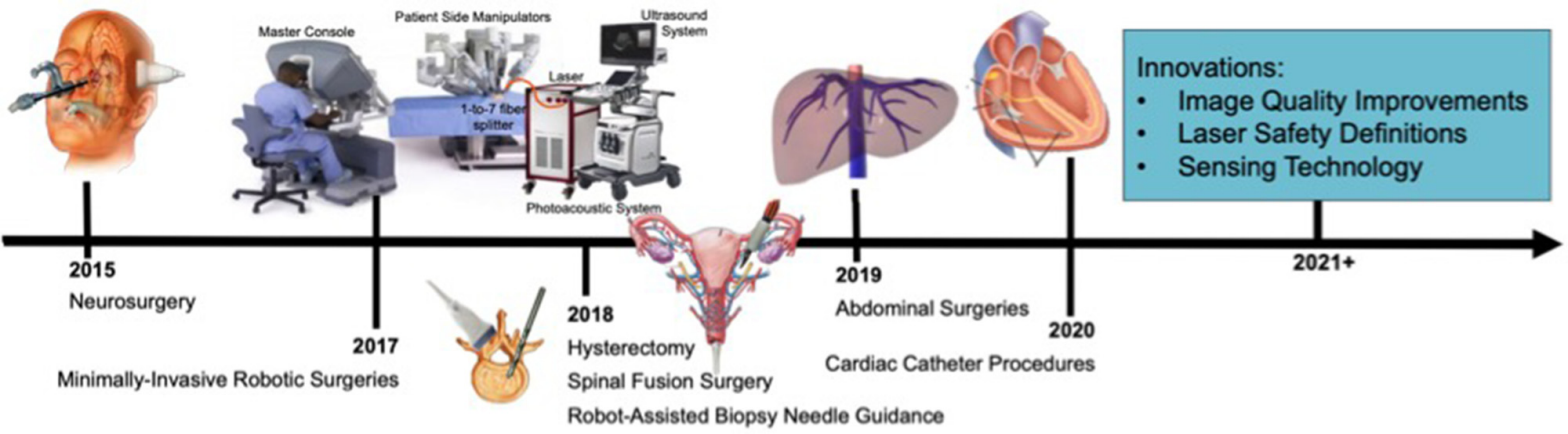
Historical perspective of PULSE Lab photoacoustic-guided surgery applications proposing light sources attached to surgical tools and integration with robotics (Sec. II), which provides support and context for the author’s perspective on possible future innovations (Sec. III).

There are multiple options available to deliver light to surgical sites. Historically, light has been attached to ultrasound receivers^5^ or located at fixed distances from the receiver,^6^ as initially presented and demonstrated for sentinel lymph node detection.^5,6^ There is now growing interest in separating the light source from the receiver and attaching light sources to surgical tools to enable guidance of interventional procedures. One underlying goal of this approach is to visualize both the tool tip and a structure that needs to be either targeted or avoided in the same photoacoustic image, as illustrated in Fig. 1. Some of the first examples of this concept were demonstrated to visualize biopsy targets such as tumors,^7^ to identify vasculature and nerves during interventional procedures,^8^ and to detect brachytherapy seeds by inserting an optical fiber through the hollow core of brachytherapy needles.^9,10^ There has since been additional work to expand these examples to other types of procedures, making the concept of photoacoustic-guided surgery more versatile than initially envisioned, presented, and demonstrated. Multiple comprehensive review articles have been written to detail the history and wide scope of photoacoustic-guided surgery applications.^2,11–13^ The purpose of this invited Perspective is to first summarize recent applications of photoacoustic-guided surgery and specific technology developed by the Photoacoustic & Ultrasonic Systems Engineering (PULSE) Lab to enable demonstrations of feasibility. This summary is organized by specific surgical and interventional challenges that are addressed using methods to avoid blood vessels, followed by methods with blood as the surgical target, followed by integration with robotic technology. Much of the recent work in this area is interconnected through the technology, equipment, and customized light delivery design innovations implemented within the past five years, as summarized in Fig. 2, which also provides a technological overview of Sec. II. The description of surgical and interventional applications in Sec. II is then followed by a discussion of outstanding challenges and opportunities in Sec. III. This Perspective concludes with a summary and outlook in Sec. IV.

## II. SURGICAL AND INTERVENTIONAL APPLICATIONS

### A. Minimally invasive neurosurgery

**FIG. 3.**
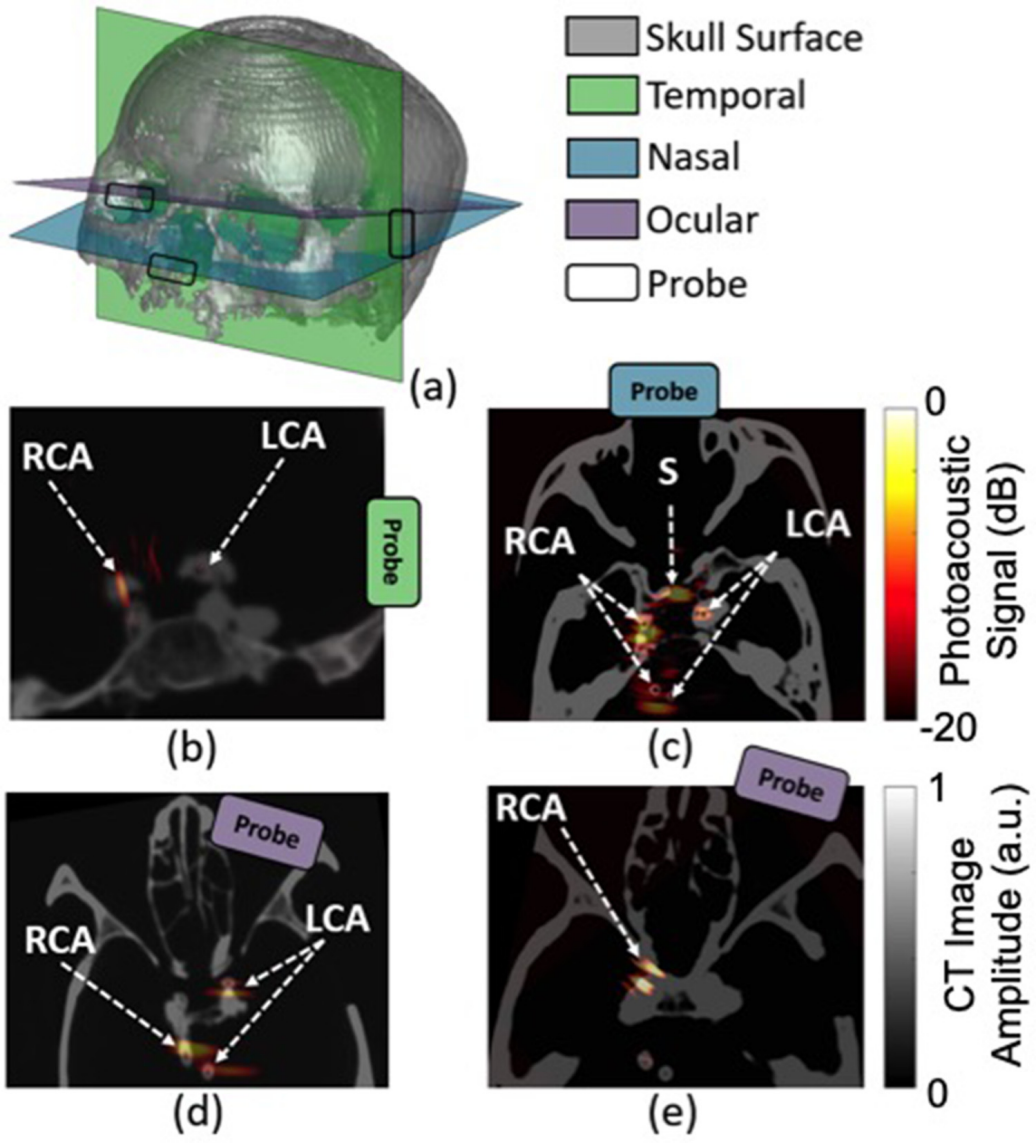
(a) 3D computed tomography (CT) skull reconstruction highlighting the axial–lateral imaging planes and lateral-elevation ultrasound probe locations used for the temporal, nasal, and ocular external ultrasound probe locations. Co-registered CT and photoacoustic images acquired with the ultrasound probe placed on the (b) temple, (c) nasal, and (d) ocular regions. (e) Co-registered CT and photoacoustic images of a skull filled with brain tissues and ovine eyes and ultrasound probe placed on the right ocular region. Photoacoustic targets include the left and right internal carotid arteries (LCA and RCA, respectively) and optical source (S). Reprinted with permission from Graham *et al*., Photoacoustics **19**, 100183 (2020). Copyright 2020 Elsevier.

Pituitary tumors located at the base of the skull are commonly removed with endonasal, transsphenoidal surgery, implemented by passing surgical tools through the nose and nasal septum and removing the sphenoid bone to access the tumor. Although the surgery is generally safe, one of the most significant complications is caused by the location of the carotid arteries within 1–7 mm of the pituitary gland, with reported incidence rates of 1%–1.1%.^15,16^ Accidental injury to these carotid arteries is a serious surgical setback that causes extreme blood loss, neurological deficits, stroke, and most significantly, the possibility of death, with a reported 24%–40% mortality rate each time this injury occurs.^15,17^ One reason mortality rates are so high is because current procedures rely on preoperative MRI or CT images, which are not updated during surgery. The overall vision to address these challenges with photoacoustic imaging is to attach optical fibers to the surgical drill^18^ and detect photoacoustic signals with an external ultrasound probe. Initially, the temple was considered as a viable ultrasound probe location,^19^ as illustrated in Fig. 2. Follow-up simulation and cadaver studies proposed the eye and the nose as alternative locations for ultrasound signal reception.^14^ Blood vessels located behind bone were successfully visualized in a human cadaver head with these external acoustic receiver locations, demonstrating the feasibility of photoacoustic-guided neurosurgery with internal light delivery and multiple possible external ultrasound probe locations, as shown in Fig. 3. These results are additionally promising for other types of transcranial photoacoustic imaging applications.

### B. Liver surgery

**FIG. 4.**
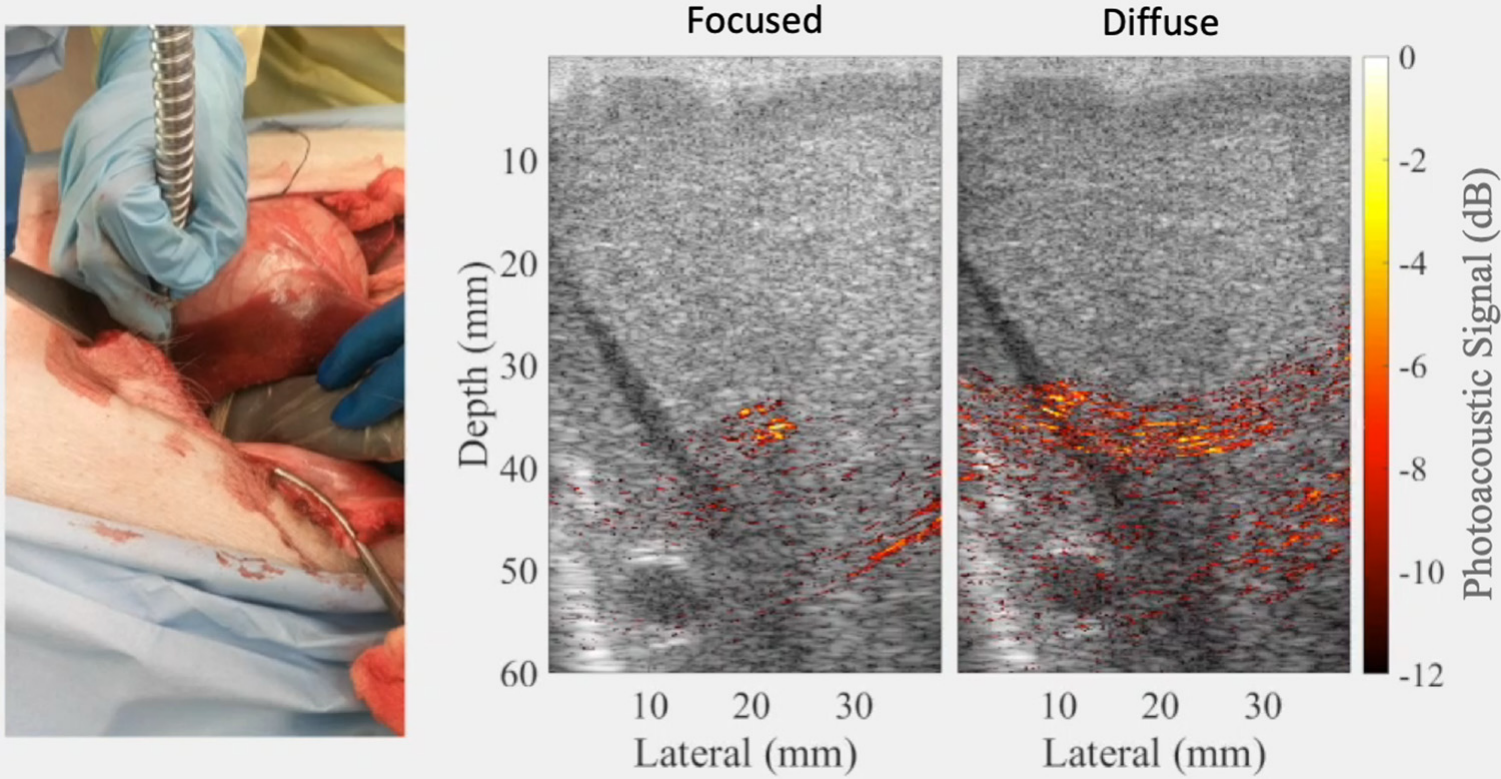
Example of photoacoustic signals within and nearby a major hepatic vein obtained during an *in vivo* liver surgery. The signals originating from within the vein had a more focused appearance, while a subtle adjustment of the light source caused more diffuse signals. Adapted from Kempski *et al*., Proc. SPIE **10878**, 108782T (2019). Copyright 2019 Author(s), licensed under a Creative Commons Attribution 4.0 License.

Liver surgeries suffer from the risk of gastrointestinal and intra-abdominal hemorrhage,^21–23^ with postoperative morbidity occurring in 23%–46% of patients who hemorrhage during liver resections and death occurring in 4%–5% of these patients.^23^ To address these complications, photoacoustic imaging was implemented by adjusting the location of the light source to determine the location of a major hepatic blood vessel based on its appearance as a focused signal, rather than a diffuse signal (which tends to indicate the presence of a predominantly liver tissue).^20^ The diffuse signal in the liver tissue was likely caused by the presence of multiple small blood vessels within the liver tissue that are either located in the image plane or located off-axis from the image plane. Examples of the differentiation between diffuse and focused signals are shown in Fig. 4, with a corollary video demonstration published in the corresponding journal publication on this topic.^20^ This differentiation, which was not present during *ex vivo* liver experiments, has two immediate implications for photoacoustic-guided liver surgeries. First, the distinguishable visualization of major blood vessels during surgery can possibly help surgeons to navigate around these vessels during tissue resection procedures. Second, this approach can be used to assist with estimating and targeting areas where cauterization of the major blood vessels is necessary in order to reduce significant bleeding and blood loss during surgery. Therefore, this is an example of the use of the proposed technology to both target and avoid blood vessels during surgery.

### C. Spinal fusion surgery

Spinal fusion surgeries are performed to alleviate pain or neurologic deficit or to repair damaged vertebrae within the spinal column^25,26^ by placing screws through the pedicles of vertebrae to connect them with a metal rod and stabilize the spine. The pedicles consist of a cancellous core that is targeted for screw insertion. However, the pedicle wall is breached in 4%–12% of procedures,^27,28^ and this rate increases up to 29% for residents.^29^ In addition to potential breaches, it is difficult to determine the optimal starting point without some initial removement of bone, which can weaken the remaining bone needed to support pedicle screw insertion, particularly if the initial position is incorrect and adjustments are required. Approximately 14%–39.8% of screws are misplaced during spinal fusion surgeries,^30–33^ which can lead to postoperative neurological injuries, vascular injuries, blindness, or necessary reoperations.^34–36^ Photoacoustic imaging has been explored as an option to uncover expected differences between the cortical and cancellous bone for the potential guidance of spinal fusion surgery.^24^ An optical fiber that delivers laser light could either be isolated from or attached to the surgical tool.^37^ A standard clinical ultrasound probe would then be placed with acoustic coupling gel on the vertebra of interest. The purpose of this ultrasound probe is to receive the acoustic response generated by optical absorption within the blood-rich cancellous core, noting that it is possible for the resulting acoustic response to travel through the 244 μm to 1.75 mm-thick cortical bone layer covering the cancellous core.^38–40^

**FIG. 5.**
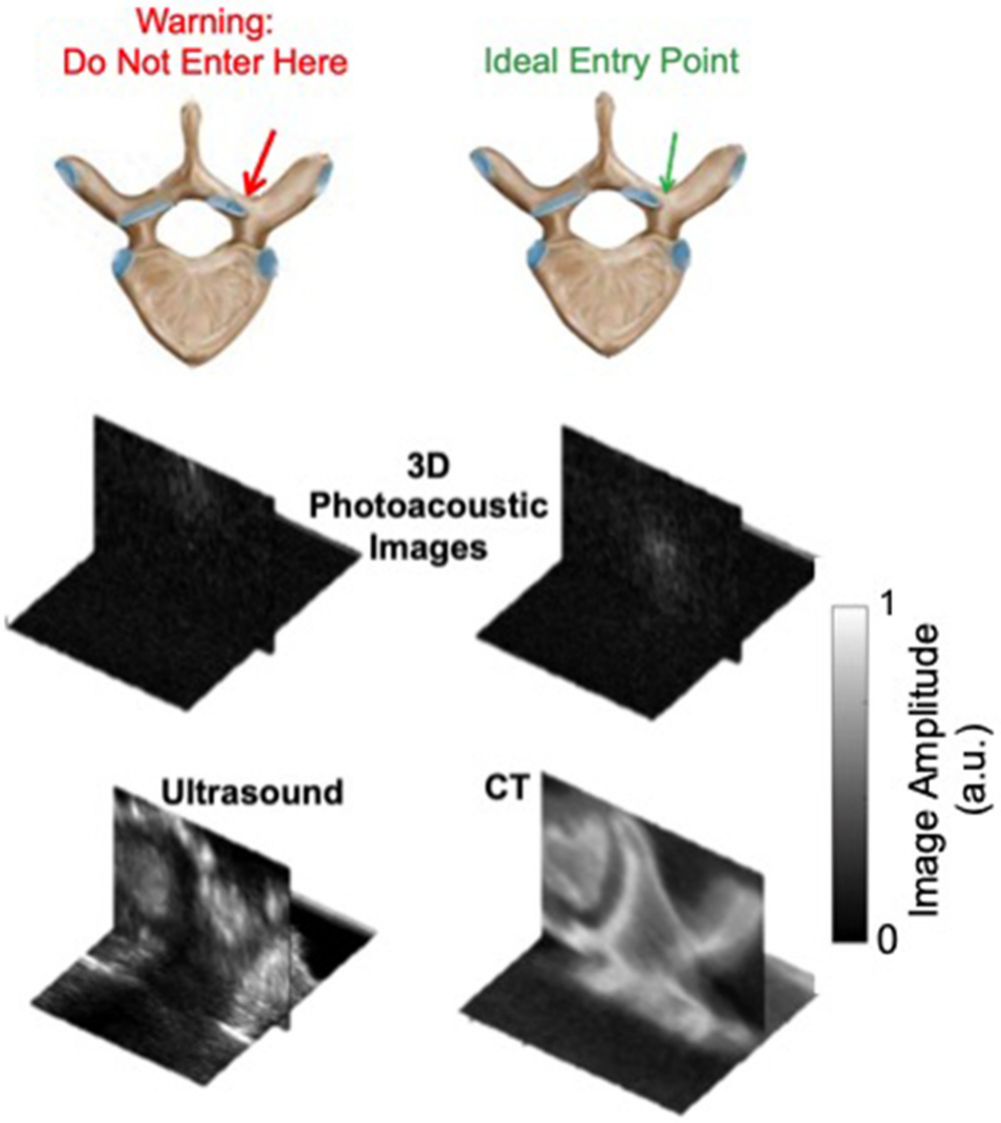
Photoacoustic signals from cortical bone (left) and the cancellous core of the pedicle (right) and corresponding ultrasound and CT volumes confirming signal source locations. Photoacoustic imaging has the potential to target the blood-rich cancellous core to assist with pedicle screw insertion during spinal fusion surgeries. Adapted from J. Shubert and M. A. L. Bell, Phys. Med. Biol. **63**(14), 144001 (2018). Copyright 2018 Author(s), licensed under a Creative Commons Attribution 3.0 Unported License.

Therefore, in this application of photoacoustic imaging for surgical guidance, blood-rich regions can be targeted rather than avoided, based on the appearance of photoacoustic signals obtained with an optical fiber pointing toward the cortical bone rather than the cancellous core of the pedicle (i.e., the ideal entry point), as shown in Fig. 5. These subtle differences are achievable because the optical absorption of blood is orders of magnitude higher than that of bone, which permits a photoacoustic response from the bood-rich cancellous core. These results are additionally insightful for other applications that involve photoacoustic imaging of (or within) bony tissue.

### D. Gynecological surgery

Approximately 52%–82% of iatrogenic injuries to the ureter occur during gynecologic surgery, often caused by clamping, clipping, or cauterizing the uterine arteries as they overlap the ureter,^41^ which are located within a few millimeters of the uterine artery (see Fig. 2). Ideally, this injury would be avoided altogether, and if it occurs, it would be best to notice it during the operation in order to address it immediately. Yet, 50%–70% of ureteral injuries are undetected during surgery,^42^ leading to multiple post-operative complications, including kidney failure and death. Photoacoustic imaging is one viable solution to detect hidden blood vessels in real time during minimally invasive gynecological surgeries and simultaneously differentiate these vessels from the ureter using photoacoustic imaging.^43,44^ Blood vessels may be distinguished from the ureter by introducing wavelength-dependent contrast agents into the urinary tract. The feasibility of this concept has thus far been explored for photoacoustic-guided hysterectomy.^43–45^ Results demonstrate that there is promise to use this technology to identify the uterine arteries and differentiate them from the ureter with the assistance of contrast agents such as FDA-approved methylene blue. This contrast agent may be administered intravenously during surgery and is required for ureter visualization due to the relatively low optical absorption of urine. Other possible gynecological applications of this technique include endometriosis resection and myomectomy (i.e., removal of uterine fibroids).

### E. Robot-assisted biopsy guidance

Biopsies are widely performed procedures, implemented by inserting a hollow core needle to extract a small piece of abnormal or suspicious tissue for examination under a microscope to make a diagnosis. While the procedure is generally effective when guided by ultrasound, it is often difficult to localize needle tips in some patients (including obese and overweight patients) due to poor ultrasound image quality and difficulty differentiating a needle tip from a needle midsection. In addition, obesity increases the risk of complications from 4% (1 insertion) to 14% ( >4 insertions).^47^

**FIG. 6.**
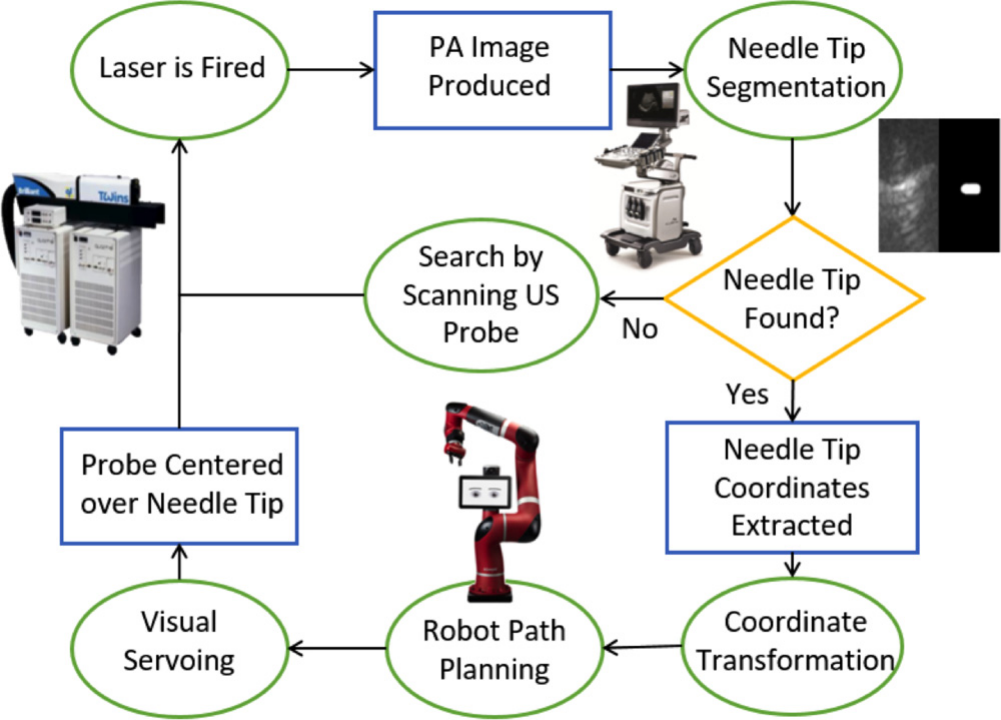
Robot-assisted photoacoustic imaging for hands-free biopsy. Reprinted with permission from J. Shubert and M. A. L. Bell, “Photoacoustic based visual servoing of needle tips to improve biopsy on obese patients,” in *IEEE International Ultrasonics Symposium* (IEEE, 2017). Copyright 2017 IEEE.

Targeting accuracy and localization of biopsy needle tips can be enhanced with photoacoustic imaging by inserting an optical fiber into the biopsy needle.^7,8^ This concept can be further enhanced by relieving operators of the burden to find photoacoustic signals of interest within the body. This relief was achieved by offering hands-free operation and commanding a robot to search, find, and stay centered on photoacoustic signals from the needle tip through a process known as visual servoing, which is vision based control, with the vision provided by photoacoustic images.^48^ This approach is illustrated in Fig. 6 and has the potential to impact multiple biopsy procedures, such as kidney, liver, and breast biopsies.

### F. Cardiac catheter-based interventions

**FIG. 7.**
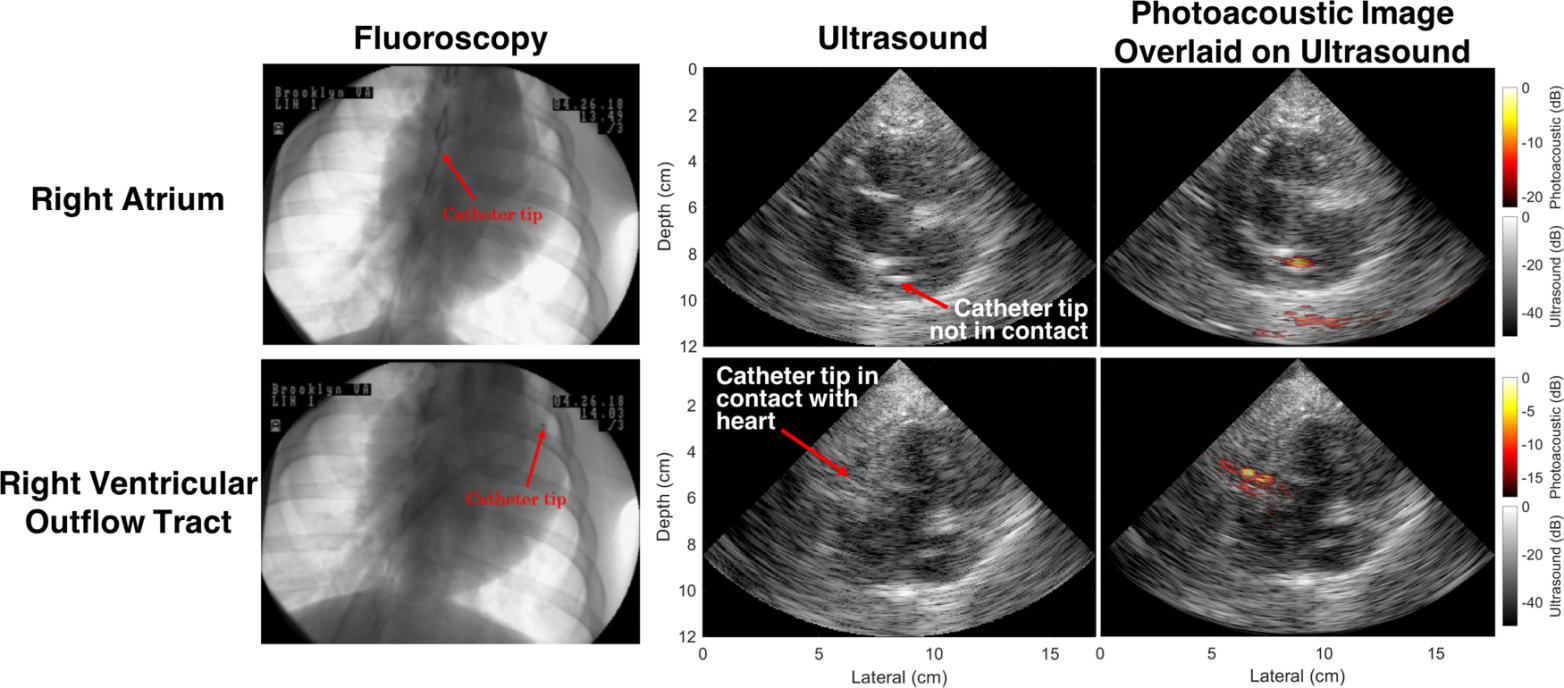
Photoacoustic signals within an *in vivo* heart, obtained with and without catheter-to-myocardium contact. Adapted from Graham *et al*., IEEE Trans. Med. Imaging **39**(4), 1015–1029 (2020). Copyright 2020 Author(s), licensed under a Creative Commons Attribution 4.0 License.

Cardiac radiofrequency ablation is the most effective treatment for atrial fibrillation (a heart disease claiming over 6.1 $\times 10^{6}$ lives). Although guided by real-time, x-ray fluoroscopy, lesion formations are not well characterized intraoperatively, which often results in repeat surgeries. Several papers report options to differentiate ablated lesions from normal tissue using photoacoustic imaging,^49^ demonstrating convincing potential to identify differences in real time^50^ and in an *ex vivo* beating heart.^51^ This potential was later expanded by proposing a full end-to-end system for replacing (or significantly mitigating) fluoroscopy with a robotic photoacoustic imaging system.^52^To realize this potential, a standard cardiac catheter would be modified to accommodate an optical fiber within its hollow core in order to generate photoacoustic signals from the catheter tip as it is guided toward the heart. These photoacoustic signals would be received by an external ultrasound probe. Using the same visual servoing procedures outlined in Sec. II E, a robot arm attached to the ultrasound probe can be commanded to maintain the catheter tip at the center of the photoacoustic image.^52^ Figure 7 demonstrates the benefit of relying on this method to identify catheter tips with more certainty than that provided by ultrasound images (and with the inclusion of depth information that is absent from fluoroscopy), particularly when the tip of the fiber-catheter pair is in contact with the myocardial tissue. In addition, the contrast differences between photoacoustic signals obtained with and without the catheter touching the endocardium can be used to confirm catheter-tissue contact prior to ablation.^52^

### G. Teleoperated robotic surgery

A range of surgeries (such as the gynecological procedures described in Sec. II D) are trending toward incorporating robotic assistance, due to the promise of decreased hospital stays, minimal blood loss, and shorter recovery periods. These robotic systems additionally have the potential to benefit from augmentation with photoacoustic imaging systems.^53^ In addition to blood vessel visualization during these minimally invasive robotic surgeries, one additional challenge that can be addressed with photoacoustic imaging is the rise of ureteral injuries with the introduction of these robots. For example, one study documented a 2.4% rate of ureter injury during pelvic surgeries performed before robotic assistance, compared with 11.4% after the implementation of the technology.^54^ With regard to robotic hysterectomies, the da Vinci® robot is the only option available for performing robotic hysterectomies, and hysterectomies are the most popular surgery for the da Vinci® robot. Therefore, it would be impactful to introduce this technology for hysterectomies.

**FIG. 8.**
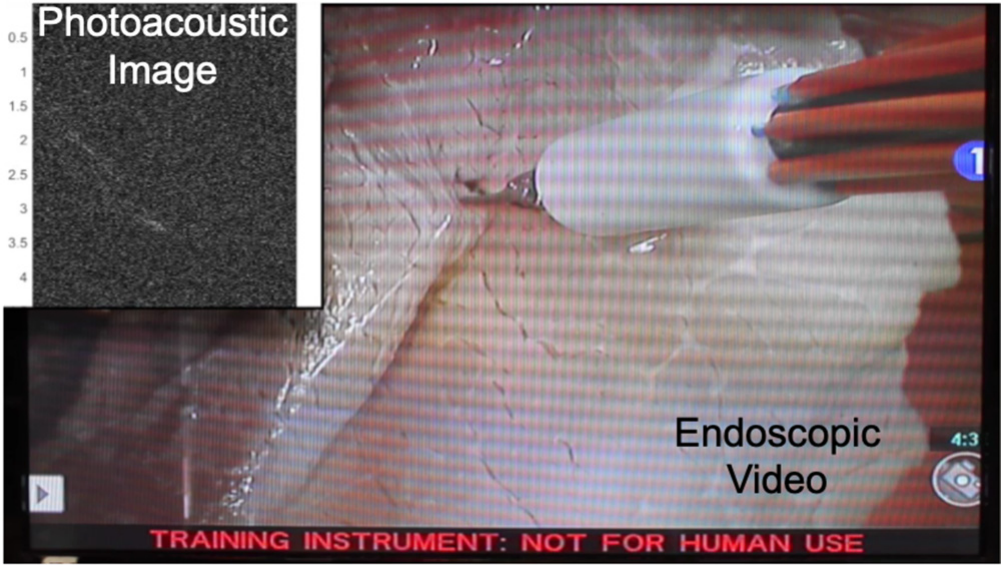
Photoacoustic signals and the corresponding da Vinci^®^ S endoscope video feed showing the surgeon’s view from the master console. Adapted from Allard *et al*., J. Med. Imaging **5**(2), 021213 (2018). Copyright 2018 Author(s), licensed under a Creative Commons Attribution 3.0 Unported License.

Toward this end, proof-of-concept experiments were performed in a mock operating room that contained a da Vinci® S robot, consisting of a master console (shown in Fig. 2), patient side manipulators that are teleoperated from the master console, and an endoscope to visualize the surgical field. A photoacoustic imaging system was positioned next to this surgical robot. To demonstrate initial feasibility,^43^ an experimental phantom was placed on a mock operating table located in a mock operating room that housed the da Vinci® robot. The integrated photoacoustic imaging system consisted of an Alpinion ECUBE 12R ultrasound system connected to an Alpinion L3-8 linear transducer (which has a 5 MHz center frequency to allow deep acoustic penetration for the received photoacoustic sound waves) and a Phocus Mobile laser with a 1-to-7 fiber splitter attached to the output port of the laser. The seven output fibers of the light delivery system surrounded a da Vinci® curved scissor tool and were held in place with a custom-designed, 3D printed fiber holder.^43^ The da Vinci® scissor tool was held by one of the patient side manipulators of the da Vinci® S robot. An example view of the resulting photoacoustic image is shown in Fig. 8, synchronized with the motion of the customized da Vinci® scissor tool, as viewed through the master console. A corollary video demonstration is published with the corresponding journal publication on this topic.^43^ The view in Fig. 8 represents one possible presentation format for surgeons during an operation. In these feasibility studies, the surgical tool and attached light delivery system was teleoperated. However, taking a broader perspective of the possibilities for robotic-photoacoustic integration, either the ultrasound probe or the fiber (or both) can be controlled with automated, semi-automated, cooperative, or teleoperative control. For example, Moradi *et al.*^55^ demonstrated the use of virtual fixtures to constrain ultrasound probe motion when implementing photoacoustic imaging with a da Vinci® research kit.^56^ These and other possibilities are not limited to the da Vinci® robot and may be implemented with a wide range of commercial or custom robots that offer the desired control capabilities.

## III. CHALLENGES AND OPPORTUNITIES

### A. Image quality

One of the major underlying and pervasive challenges with photoacoustic imaging approaches that rely on low-frequency ultrasound probes, which are ideal for deep acoustic penetration during surgery, is that these lower frequencies tend to produce relatively poor image quality (as opposed to the clearly differentiated vasculature that has historically been observed with high-frequency, microscopic photoacoustic techniques). Metrics to assess image quality should always be tied to the imaging task at hand, with common objective metrics for surgical guidance concerned with target visibility, sizing, and location accuracy, as defined by contrast, resolution, signal-to-noise ratio, and contrast-to-noise ratio. Considering the typical trade-off between these image quality metrics and the important advantage of offering deep acoustic penetration, low-frequency ultrasound probes are arguably the best candidates for surgical guidance with internal light delivery and external sound reception at large depths from the target location, provided that the desired specifications, image quality metrics, and sensitivity requirements are met. Therefore, methods to improve image quality would benefit from focusing on viable options that are compatible with the technical capabilities of low-frequency ultrasound probes.

**FIG. 9.**
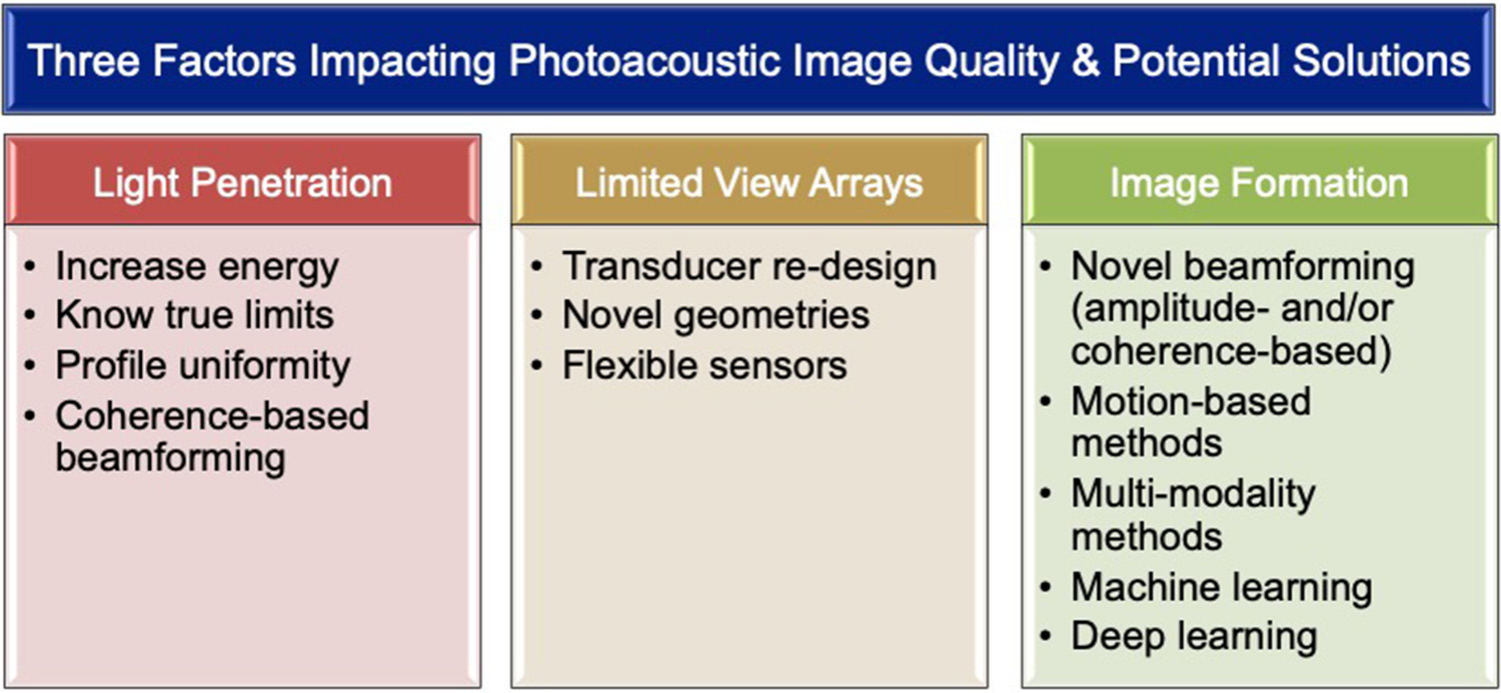
Three key factors impacting photoacoustic image quality for surgical guidance (top) and possible solutions to address challenges (bottom).

Three notable factors (excluding ultrasound transducer frequencies for the reasons stated above) that primarily impact photoacoustic image quality for surgical guidance tasks are summarized in Fig. 9: (1) limited light penetration, (2) “limited-view” linear/phased/curvilinear array ultrasound sensors (in comparison to ring arrays), and (3) image formation and beamforming models that do not consider multipath acoustic propagation. Figure 9 also includes a summary of some potential solutions, with details on possible methods to address the first two factors discussed in Secs. III B and III C, while a discussion of the third factor is the focus of this section. In particular, artifacts introduced by multiple acoustic pathways are common to both ultrasound and photoacoustic imaging, producing what is known as acoustic clutter,^48,57^ with larger illumination areas expected to produce more photoacoustic clutter. The simplest attempts to address acoustic clutter and related image quality challenges include post-processing to clean-up photoacoustic images and thresholding of these images prior to display. In addition, while research effort has been dedicated to addressing these challenges from an optics perspective,^58^ this challenge has primarily been framed from an acoustics perspective. Promising acoustic-based approaches include frequency based methods,^59,60^ mixed imaging modality methods,^61–64^ motion-based methods,^64–66^ and advanced beamforming techniques, such as adaptive beamforming^67^ or short-lag spatial coherence beamforming,^10,68–71^ which was recently implemented in real-time for photoacoustic-based visual servoing.^72–74^ With both simple and complex methods to improve image quality, it would be prudent to re-evaluate our methods to quantify image quality, as recently demonstrated with the relatively new metric of a generalized contrast-to-noise ratio^75^ applied to photoacoustic images.^76^

Another active area of recent interest and investigation to improve image quality is the integration of modern machine learning techniques with image formation.^77–86^ Specifically, deep learning alternatives to image formation have been used to replace either a subset of or the entire mathematical component of image formation with a well-trained model or a neural network. The use of advanced beamformers and the selection of deep neural networks for image formation should ideally be based on the specific task at hand. In addition, implementations of these methods with the same ultimate goal of improved image quality should ideally be as simple as possible, with increasing levels of complexity added only as absolutely necessary. This philosophy often results in a trade-off between image quality and real-time implementation speed, which are often inversely related.

Based on the perspective that deep learning has strong potential to be the latest frontier that optimizes the trade-off between image quality and speed, deep learning is particularly promising for image-guided surgery because unique acoustic signatures can be learned with attention to light delivery systems that are fashioned around surgical tool tips. For example, one option is to learn the unique shape-to-depth relationship of point-like photoacoustic sources from metallic surgical tool tips and nearby point-like structures in order to provide a deep learning-based replacement to common photoacoustic image formation steps.^78,83^ This approach has the potential to be extended to other structures with unique photoacoustic signatures.

### B. Laser safety and energy requirements

**FIG. 10.**
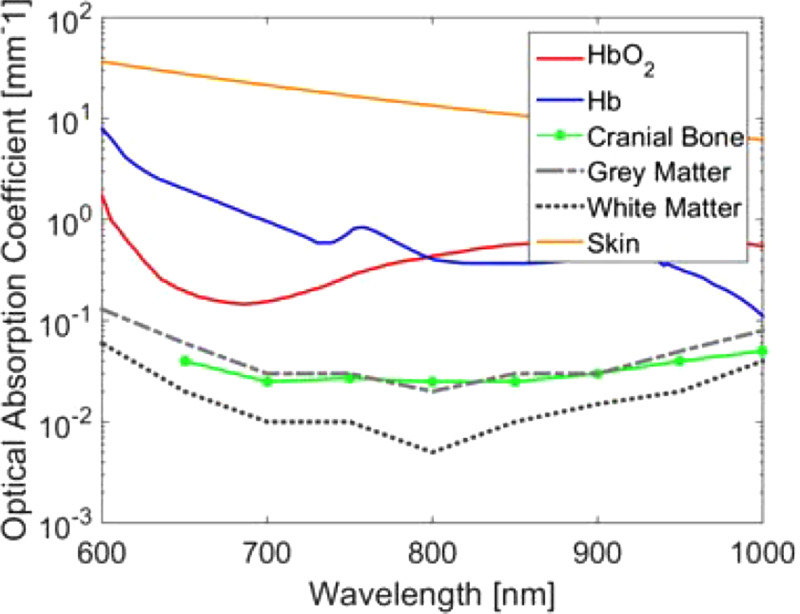
Optical absorption of blood, bone, brain tissues, and skin.^90–93^

When implementing any photoacoustic-guided surgery or interventional application, it is critically important to consider the acceptable laser exposure for the surrounding tissue. If energy exposure is too great, tissue may coagulate or vaporize, which may not be desirable depending on the specific surgery. In general, laser radiation causes thermal effects depending on the wavelength, power, energy, beam diameter, and absorption spectrum of the tissue. Additional considerations include the volume of circulating blood, specific heat, thermal conductivity, non-homogeneity, and optical properties such as transmission, reflection, absorption, and scattering.^87^ The maximum permissible exposure (MPE) is defined by the American National Standards Institute as the level of laser radiation to which an unprotected person may be exposed without adverse biological changes in the eye or skin.^88^ Based on this definition, there is no standardized tissue-specific limit (e.g., for brain, prostate, liver, or heart tissue), and therefore, the photoacoustic community generally assumes that the MPE for skin is an acceptable baseline. While the community often acknowledges that this is a rather conservative limit for multiple reasons,^8,9,89^ the following text provides an additional perspective for brain tissues and neurosurgery that can be applied to many other tissues and surgical (or interventional) applications. Figure 10 demonstrates that brain tissue (i.e., white and gray matter) has a lower optical absorption than melanin and collagen, which are major structural components in skin.^92,93^ This difference indicates that the brain tissue is likely to absorb less energy compared to skin for any given applied energy. Additionally, the brain tissue has a specific heat of 3630 J/kg/°C, whereas skin has a specific heat of 3391 J/kg/°C,^94^ indicating that for an equal amount of absorbed energy, the temperature will be raised more in skin than in the brain tissue (assuming equal unit mass). Normal body temperature is approximately 37 °C with up to 1 °C variations. Brain damage occurs above 42 °C and skin damage occurs over 44 °C. Thus, the MPE for skin is a rather conservative value.

When imaging with lasers, the MPE depends on a variety of factors such as wavelength, λ, and exposure duration. For λ=700–1050 nm and an exposure duration of 10^9^ s (which is standard for photoacoustic imaging), the MPE equation for skin is^88^

$$MPE=2.0(10^{0.002(\lambda -700)})\cdot 10^{-2}\;\left[J/cm^{2}\right].$$ (1)

Considering that exposure is defined per 1 cm^2^ unit, MPE comparisons can be approached by analyzing a 1 cm^2^ area of skin. Taking the average thickness of skin to be ≈2 mm^95^ results in a hypothetical volume of 0.2 cm^3^. Given that the body is comprised largely of water (density = 1 g/cm^3^), the 0.2 cm^3^ volume is assumed to correspond to a mass of ≈0.2 g. The MPE at 760 nm [i.e., 26.4 mJ/cm^2^ based on Eq. (1)] can then be expressed as (26.4 mJ)/(0.2 g) and converted using specific heat values to the following temperature rises for both skin and brain:

$$\frac{26.4\;\mathrm{mJ}}{0.2\;g}\cdot \frac{1\;\mathrm{kg}\;{}^{\circ}C}{3391\;J}=0.039\;{}^{\circ}C\;\left(\mathrm{skin}\right),$$ (2)

$$\frac{26.4\;\mathrm{mJ}}{0.2\;g}\cdot \frac{1\;\mathrm{kg}\;{}^{\circ}C}{3630\;J}=0.036\;{}^{\circ}C\;\left(\mathrm{brain}\right).$$ (3)

Assuming that 100% of the applied energy is absorbed (which is not the case), the change in the temperature for brain would be lower than that of skin, and both new temperatures would be within 1 °C of standard body temperature. These calculations suggest that MPE is a very conservative value for the brain tissue, as the maximum exposure is expected to raise temperature by only a fraction of a degree, and furthermore, only mK temperature rises are required for photoacoustic imaging. Note that this analysis does not consider potential differences in fluence profiles, particularly that the peak temperature is expected to be larger where the light first emerges from the fiber, thereby giving rise to a larger peak temperature rise than the average, which highlights the importance of developing uniform light profiles for the proposed technologies.^18^ Similarly, small blood vessels could experience larger local temperature rises in the brain or the skin tissue (depending on the excitation wavelength), and this additional consideration is expected to similarly impact both tissue types. Therefore, the presented estimates are expected to be conservative regardless of the inclusion of this additional consideration, and this conservativeness often comes at the expense of poor signal-to-noise ratios, which is one of the metrics of poor image quality noted in Sec. III A. The ability to increase laser energies for specific tissues beyond the existing limits for skin enables the improvement of poor image quality and target differentiation caused by limited light penetration.^9,20,52^

In addition to defining the maximum energy limits, it is also important to define the minimum energy requirements. Although the minimum required energy is expected to partially depend on the overall imaging system design (and this energy limit will likely be different for different photoacoustic system configurations), the minimum required energy can potentially be generalized based on the imaging environment. For example, imaging through bone is expected to require higher minimum energies than imaging through soft tissues, with all else being equal or at least similar (e.g., receiver sensitivity, beamforming method, image post-processing steps). This information will be useful to finalize novel system design and performance requirements, as the minimum required energy is one component of the overall system design that determines which type of light source is truly needed (e.g., traditional Q-switched lasers, alternative options discussed in Sec. III C, new light sources to be determined, introduced, or invented based on the knowledge of true input energy limits for a specific surgical or interventional application).

In particular, when navigating to specific targets using a combination of photoacoustic imaging and robotics (e.g., visual servoing of photoacoustic signals from a needle tip to improve the biopsy of obese patients^46^), one critical step of this procedure is the segmentation algorithm used to define the tip location coordinates for robot path planning, as summarized in Fig. 6. Ideally, the minimum energy required for fiber tip localization would be used. Understanding this minimum required energy for the robot to perform its visual servoing task would ensure that minimum energies are used without the empirical testing period that has preceded previous *in vivo* animal experiments,^52^ which is not ideal for operation within the human body. If this understanding of minimum energy requirements can be developed based on the theoretical knowledge of signal and image quality limitations, then the associated theoretical equations can replace empirical testing with evidence-based predictions. This information about minimum input energy requirements would then be implemented without requiring the time- and resource-intensive experiments currently needed to test multiple possible system configurations prior to initiating an interventional procedure.

### C. Alternative imaging and sensing technology

Ideal acoustic sensors for guiding surgery would be small, compact, and flexible. These properties are generally uncharacteristic of current clinical and pre-clinical sensors that are used for photoacoustic imaging in other applications. For example, demonstrations of photoacoustic image guidance of the *in vivo* liver^20^ would benefit from a reduction in the ultrasound probe size to avoid interference with the tight surgical workspace (with an expected trade-off between the array size and the lateral resolution). Photoacoustic-based surgical guidance during neurosurgery would similarly benefit from a smaller, customized nasal ultrasound probe with a water-filled, balloon tip for acoustic coupling.^14^ Another promising and upcoming technology is the utilization of flexible ultrasound sensors^96,97^ for surgical guidance. These sensors have the potential to conform to the various shapes and sizes of multiple organs during surgery, thereby making the sensors as unobtrusive as possible. Flexible sensors can also be contorted to shapes that partially address the limited view-related image quality challenges noted in Sec. III A. These flexible sensors pose a new set of beamforming challenges as well, considering that the array geometry is expected to change frequently with this newfound flexibility. Wireless ultrasound signal transmission^98^ is yet another area that could be used to improve photoacoustic image guidance.

Alternative light sources are one additional innovative direction for the future of photoacoustic imaging to guide surgeries. There has been a growing interest in pulsed-laser diodes^99,100^ and light emitting diodes (LEDs)^101,102^ as light sources for photoacoustic imaging. Each alternative has benefits, such as lower power, smaller, more portability, and higher frame rates. Incidentally, one of the primary benefits of these alternative light sources is also one of the greatest challenges with this alternative. In particular, the lower energies of these light delivery methods produce low signal-to-noise ratios that are not suitable for deep optical penetration, often resulting in the requirement for signal averaging to improve image quality, which slows down the critical frame rates that are needed for real-time guidance (which can be overcome with coherence-based beamforming if target location information without amplitude information will suffice,^103^ such as in visual servoing tasks^74^). In addition, multiple LEDs are currently required to deliver signal quality that is on par with larger laser systems, resulting in bulkier light delivery systems that are not suitable for surgical guidance or attachment to the tips of surgical tools.

### D. Clinical translation

The ultimate goal with much of the efforts described within this Perspective is to translate the described technology for the benefit of patients. In addition to the associated translational challenges and possible future directions described above and summarized in Fig. 9 (e.g., image quality improvement solutions, laser safety and energy requirements, light delivery, and sensing technology considerations), the potential for clinical adoption presents an additional set of considerations (summarized in Table I). These considerations include the development of viable phantoms for technique standardization among hospitals, training of surgeons and their assistants to interpret potentially confusing photoacoustic images, and controlling the increased flexibility introduced by separating the optical delivery from the acoustic reception to ensure that the target of interest is truly identified and sufficiently exists within the tomographic imaging plane as intended. This last detail will minimize image misinterpretation from out-of-plane photoacoustic signals.^43^

**TABLE I.** Summary of important requirements and considerations for surgical and interventional translation of promising photoacoustic imaging methods and applications.

Requirements	Considerations
Small/portable systems	Image quality
	Reduced input energies
	Reduced lateral resolution
	Possibilities for wireless systems
	Overall miniaturization
Real-time images	Transmit pulse repetition frequencies
	Complexity of image formation software
	Image fidelity to detected structures
Usable form factors	System components tailored to anatomy (e.g., nose, eyes)
	Integration with medical robots
Repeatable training methods	Phantom standardization across hospitals
	Imaging plane navigation
	Image interpretation
Reasonable cost/complexity	Balance with technological benefits
Regulatory clearance	Sterilization protocols
	FDA approval
	Large vs small animal testing
	Role of human cadaver testing
	Patient testing

Novel form factors that do not currently exist must also be considered, such as dual-probe systems tailored for simultaneous ocular reception of transcranial signals from both eyes of a patient.^14^ The development of photoacoustic-based, patient-specific, pre-operative path planning simulation methods is another important consideration. In addition, completely wireless photoacoustic imaging systems and overall system miniaturization are futuristic technological avenues to explore to support clinical adoption within operating rooms and interventional suites. Clinical adoption additionally requires overall ease of use, as well as system costs and complexity levels that do not outweigh technological benefits, which is particularly necessary when guidance tasks are extended to incorporate robotic assistance.

These technological challenges exist alongside complementary regulatory challenges, such as the requirement to develop dedicated sterilization protocols, the process to achieve regulatory device approval (such as FDA approval in the United States), and the need to complete pilot patient testing prior to commercialization. One intermediary step toward progress in these areas is the use of human cadavers and large animal models to “test-drive” the proposed technology. These valuable components of the technology development pipeline have proven to be more insightful than studies with small animal models (e.g., rodents) or *ex vivo* tissues. In particular, small animals often do not have the organ sizes and relative anatomical scale necessary to translate findings to humans, while *in vivo* studies with large animals can reveal trends that are simply not present in *ex vivo* and experimental phantom data (e.g., photoacoustic signal differences based on subtle positioning of the light source relative to a major hepatic blood vessel in an *in vivo* liver^20^). Similarly, new insights for photoacoustic image guidance of neurosurgery were gained by testing associated ideas with human cadaver heads and simulations.^14^ These paired simulation and human cadaver experiments validated the use of patient-specific simulations of acoustic wave propagation as a potential method to predict the optimal placement of acoustic receivers, resulting in exploration of the eye as an acceptable acoustic window for photoacoustic-guided surgeries of the skull base.^14^ These types of counter-intuitive findings are expected to be major factors that will propel the field toward translation of the proposed surgical guidance technology for widespread use by surgeons and for the benefit of patients worldwide.

## IV. SUMMARY AND OUTLOOK

There is a wide range of possible directions to pursue with regard to photoacoustic image guidance of minimally invasive surgeries and interventional procedures. Augmented surgical tools range from drills and scissors to needles and catheters. The light source can also be operated independently of surgical tools. The light source or acoustic sensor may additionally be integrated into robotic systems to enhance the overall surgical experience. Opportunities for additional advancement include image quality optimizations, redefinition of laser safety (particularly with regard to identifying tissue-specific safety limits), novel wireless and flexible sensor designs, and alternative light sources to the common Q-switched lasers. One additional promising area of opportunity that was not discussed (due to the focus on novel surgical and interventional applications and associated hardware and software innovations) is the development of new contrast agents for surgical guidance.^104,105^ Overcoming the outstanding challenges associated with the totality of these opportunities would enable widespread clinical and surgical utility. With its excellent ability to provide optical absorption contrast, acoustic penetration depths that are common to traditional ultrasound imaging, and multiple possibilities for clinical, surgical, and interventional benefit, photoacoustic imaging has much potential to become a standard technological assistant in operating rooms and interventional suites in the years ahead.

## Data Availability

Data sharing is not applicable to this article as no new data were created or analyzed in this study.
